# Motor cortex activity predicts response alternation during sensorimotor decisions

**DOI:** 10.1038/ncomms13098

**Published:** 2016-10-07

**Authors:** Anna-Antonia Pape, Markus Siegel

**Affiliations:** 1 University of Tübingen, Department CIN & MEG Center, Centre for Integrative Neuroscience & MEG Center, Otfried-Müller-Str 25, University of Tübingen, 72076 Tübingen, Germany; 2 IMPRS for Cognitive and Systems Neuroscience, Österbergstr. 3, 72072 Tübingen, Germany

## Abstract

Our actions are constantly guided by decisions based on sensory information. The motor cortex is traditionally viewed as the final output stage in this process, merely executing motor responses based on these decisions. However, it is not clear if, beyond this role, the motor cortex itself impacts response selection. Here, we report activity fluctuations over motor cortex measured using MEG, which are unrelated to choice content and predict responses to a visuomotor task seconds before decisions are made. These fluctuations are strongly influenced by the previous trial's response and predict a tendency to switch between response alternatives for consecutive decisions. This alternation behaviour depends on the size of neural signals still present from the previous response. Our results uncover a response-alternation bias in sensorimotor decision making. Furthermore, they suggest that motor cortex is more than an output stage and instead shapes response selection during sensorimotor decision making.

## Abstract (web summary)

The motor cortex executes responses based on sensory choices, but it is unknown whether it also impacts response selection. Here, Pape and Siegel show that motor cortex activity present before decision making predicts responses and that this activity is influenced by previous button-presses.

## Graphical abstract

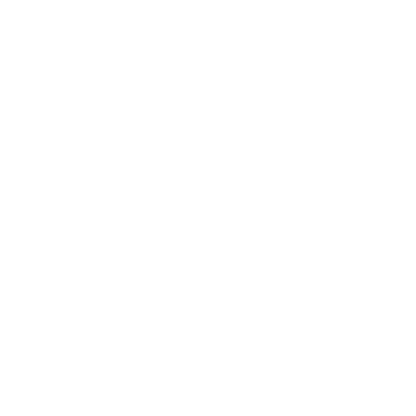

We constantly use sensory information to choose between alternative motor actions. The neural processes underlying such sensorimotor choices include the representation of sensory evidence, possibly weighing in top-down factors, deciding between choice alternatives and finally executing the appropriate motor response^1^,^2^,^3^,^4^. Traditionally, these processes were viewed as sequential stages, in which the motor cortex acts as the final output stage that merely executes responses (for example, a specific button press) corresponding to the choices made in other brain regions (for example, ‘yes—I saw the target').

In contrast to this sequential view, recent evidence suggests a more continuous flow of information and that the motor cortex, that is, primary and pre-motor cortex, is more directly involved in the decision-making process itself^4^. Before choice commitment, motor cortex activity already reflects competing response options^5^,^6^,^7^,^8^, and if choices are inextricably linked to a specific response during decision formation, activity in motor areas^6^,^9^,^10^,^11^,^12^ as well as corticospinal excitability^13^,^14^ and motor reflexes^15^ track the evolution of upcoming choices.

However, if choice–response contingencies are specified before decision making, choices and associated responses cannot be dissociated, neither behaviourally nor neurally. Therefore, it is unclear if intrinsic fluctuations of motor cortex activity have a direct impact on the decision-making process beyond representing upcoming choice-contingent responses. Here, we overcome this limitation by dissociating choices and responses, and investigate with magnetoencephalography (MEG) the motor cortex' role in human sensorimotor decision making.

We show that fluctuations over motor cortex before decision making are predictive of upcoming responses. These signal fluctuations are partly carried over from the previous response and predict a tendency to alternate between response alternatives for consecutive choices. Our results reveal a tendency to alternate responses in perceptual decision making. Furthermore, they suggest that motor cortex can impact response selection during decision making.

## Results

### Dissociating choices from responses

**Figure 1:**
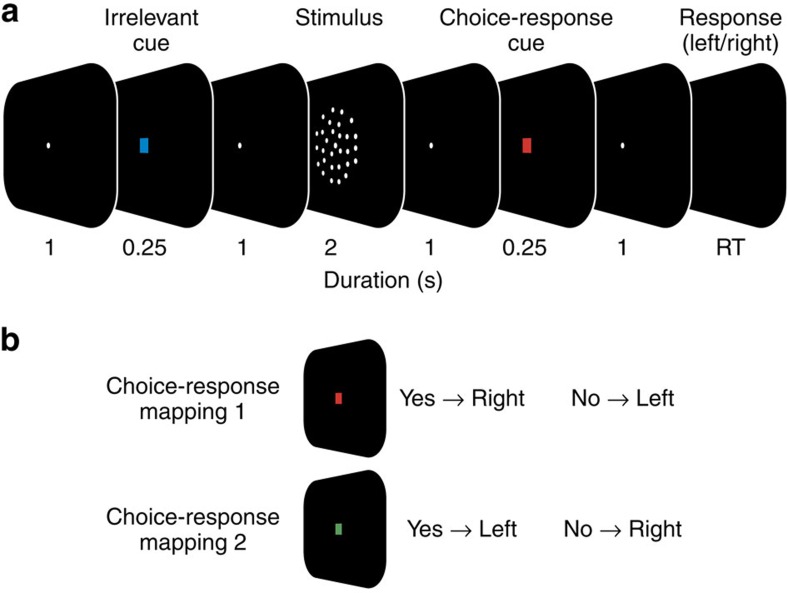
Visuomotor decision task. (**a**) Participants reported the presence of coherent motion in a display of randomly moving dots with a left- or right-hand button-press. In each trial, the mapping from choice to response hand was newly assigned with a colour cue after the stimulus (choice-response cue). Successive trials were separated by a variable length ITI (median ITI: 1,290 ms). (**b**) For a red cue (choice-response mapping 1), participants reported the presence and absence of coherent motion with a right and left hand button-press, respectively. The mapping from choice to response was reversed for the green cue (choice-response mapping 2).

We recorded MEG from 20 human participants while they judged the presence of weakly coherent motion in a display of randomly moving dots (Fig. 1a; see ‘Methods' section). For each participant, stimuli were adjusted for near-threshold performance (average correct performance: 73.9 % +/−9.4%). Subjects reported their choice (‘yes'/‘no') with a left or right hand button-press. Two design features dissociated choices from motor responses during the decision-phase^16^,^17^,^18^: First, the mapping between choice and response hand was randomly re-assigned on each trial. Second, for each trial, the choice-response mapping was indicated with a colour cue only after the stimulus presentation was completed (Fig. 1b). Thus, subjects had to form their decision during stimulus presentation, but could only later map their choice onto a response.

### Early response-predictive motor cortex activity

**Figure 2:**
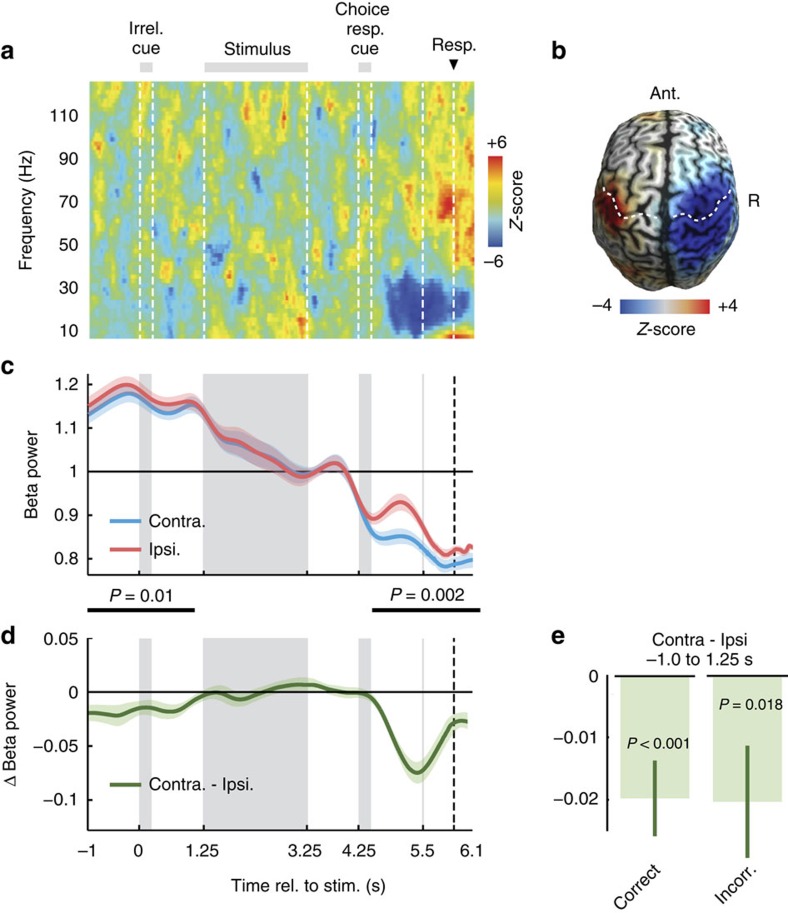
Motor cortex activity predicts upcoming responses. (**a**) Time-frequency analysis of the difference of source-reconstructed motor cortex activity contralateral minus ipsilateral to each button-press. Beta power (12–30 Hz) shows a characteristic contralateral suppression before the button-press. *Z*-scores across subjects (*n*=20 subjects). (**b**) Beta power (12–30 Hz) immediately before left minus right button-presses (4.5–5.5 s). Beta power suppression is focused on motor cortex. White dashed lines mark the central sulcus. (**c**) Time-course of beta power (12–30 Hz) in motor cortex contra- and ipsilateral to the button-press. Activity is normalized by the mean across trials. Shaded areas indicate SEM across participants. Black bars mark significant differences, that is, response-predictive activity (−1.0 to 1.1 s, *P*=0.01; 4.5–6.6 s, *P*=0.002; two-tailed one-sample cluster permutation tests, *n*=20). (**d**) Time-course of response-predictive beta activity, that is, of the difference in beta power between hemispheres contra- and ipsilateral to the button-press. (**e**) Difference between contra- and ipsilateral beta power averaged across the prestimulus period (−1 to 1.25 s) is significantly different from 0 in both correct (*P*<0.001) and incorrect trials (*P*=0.018, two-tailed one-sample permutation tests, *n*=20).

We reconstructed neuronal activity in the left and right motor cortices as a function of time and frequency (Fig. 2a). After the choice–response cue and directly preceding the button-press, we observed the typical reduction of beta-band power (12–30 Hz) in the hemisphere contralateral to the button-press (Fig. 2a, *P*=0.012, two-tailed one-sample cluster permutation test; *n*=20, 4.7–6.6 s, 10–44 Hz)^9^,^10^,^19^,^20^,^21^,^22^. Because the cortical distribution of this lateralized pre-response activity peaked pre- and post-centrally (Fig. 2b; 4.5–5.5 s; 12–30 Hz), we refer to it as sensorimotor cortex activity in the following. To test if sensorimotor cortex activity also predicted responses earlier, that is, before the choice-response cue allowed for choice-contingent response selection, we compared beta-band activity (12–30 Hz) contra- and ipsilateral to the response throughout the trial (Fig. 2c,d). This revealed significant response-predictive lateralization not only after the choice–response cue (Fig. 2c,d; 4.6–6.1 s; *P*=0.002, two-tailed one-sample cluster permutation test; *n*=20) but also at the beginning of the trial (−1.0 to 1.1 s; *P*=0.01, two-tailed one-sample cluster permutation test; *n*=20). Beta-band activity contralateral to the button-press was significantly lower than ipsilateral. This early response-predictive activity was independent of accuracy. It was present for both, correct and error trials (Fig. 2e, and Supplementary Fig. 1).

In sum, neuronal activity in sensorimotor cortex predicted which button participants eventually pressed not only after, but even before the choice-response cue, before the stimulus and more than 6 s before the final motor response. Importantly, because choices and responses were dissociated at this point in time, this response-predictive lateralization reflects neuronal encoding of the upcoming response, but not of the reported choice content.

### Long-lasting effect of beta rebound

**Figure 3:**
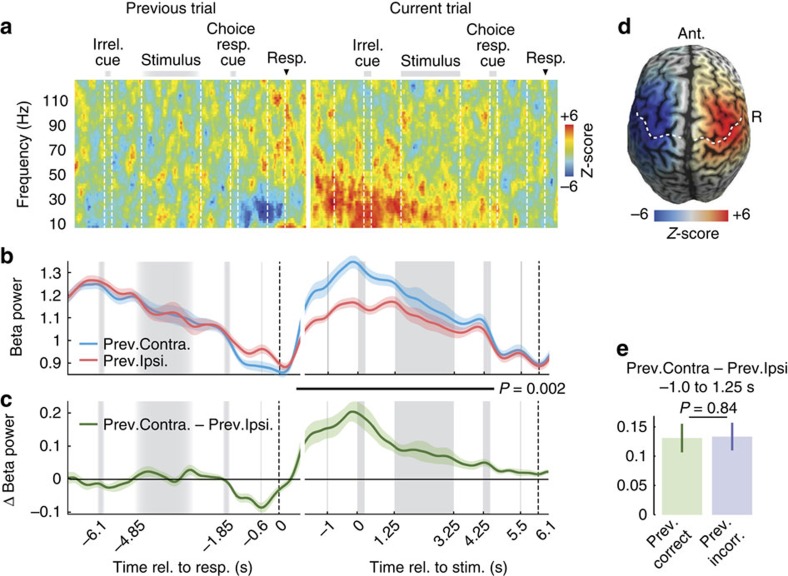
Motor signals from previous trial affect current trial. (**a**) Time-frequency analysis of motor cortex activity contralateral minus ipsilateral to previous trial's button-press in pairs of consecutive trials. Data from previous trial is aligned to button-press. Data from the current trial are aligned to stimulus onset. Data from consecutive trials are concatenated according to median ITI. *Z*-scores across subjects (*n*=20). (**b**) Time-course of beta power (12–30 Hz) in motor cortex contra- and ipsilateral to previous trial's button-press. Activity is normalized by the mean across trials. Shaded areas indicate SEM across participants. Black bar marks a significant difference from 0.7 s after the previous button-press to 4.6 s in the current trial (*P*=0.002, two-tailed one-sample cluster permutation tests, *n*=20). (**c**) Time-course of the difference in beta power contra- and ipsilateral to the previous trial's button-press. (**d**) Beta power after left minus right button-presses (−1 to 1.25 s). Dashed lines indicate the hand representation of primary motor cortex and the central sulcus, respectively. (**e**) Beta rebound averaged across the prestimulus period is not significantly different after correct and incorrect choices (*P*=0.84, two-tailed paired permutation test, *n*=20).

Because response-predictive activity appeared already at trial onset, we hypothesized that it was related to the previous trial's response. The contralateral beta power decrease in motor cortex before a response is typically followed by a characteristic increase of beta power, the ‘beta rebound'^20^,^23^,^24^. To investigate if this affected the early response-predictive activity, we analysed the evolution of the beta rebound that followed the previous trial's button-press (Fig. 3). Indeed, we found a prominent increase of beta power contralateral to, and following the previous button-press that lasted for several seconds into the current trial until presentation of the next choice- response cue (Fig. 3a–c, 0.7 s after the previous trial's button-press to 4.6 s of the current trial, *P*=0.002, two-tailed one-sample cluster permutation test; *n*=20). The cortical distribution of this beta-rebound peaked over sensorimotor cortices (Fig. 3d), and similar to the response-predictive activity, was independent of response accuracy. Furthermore, the beta-rebound did not differ following correct and error trials (Fig. 3e and Supplementary Fig. 1). At its maximum before the current trial's stimulus onset, the beta-rebound lateralization was about three times as strong as the lateralization right before the previous button-press. Thus, at the beginning of the current trial, the sensorimotor cortex was not in a neutral state, but even stronger and reversely lateralized than preceding the previous response.

### Beta rebound predicts response alternation

**Figure 4:**
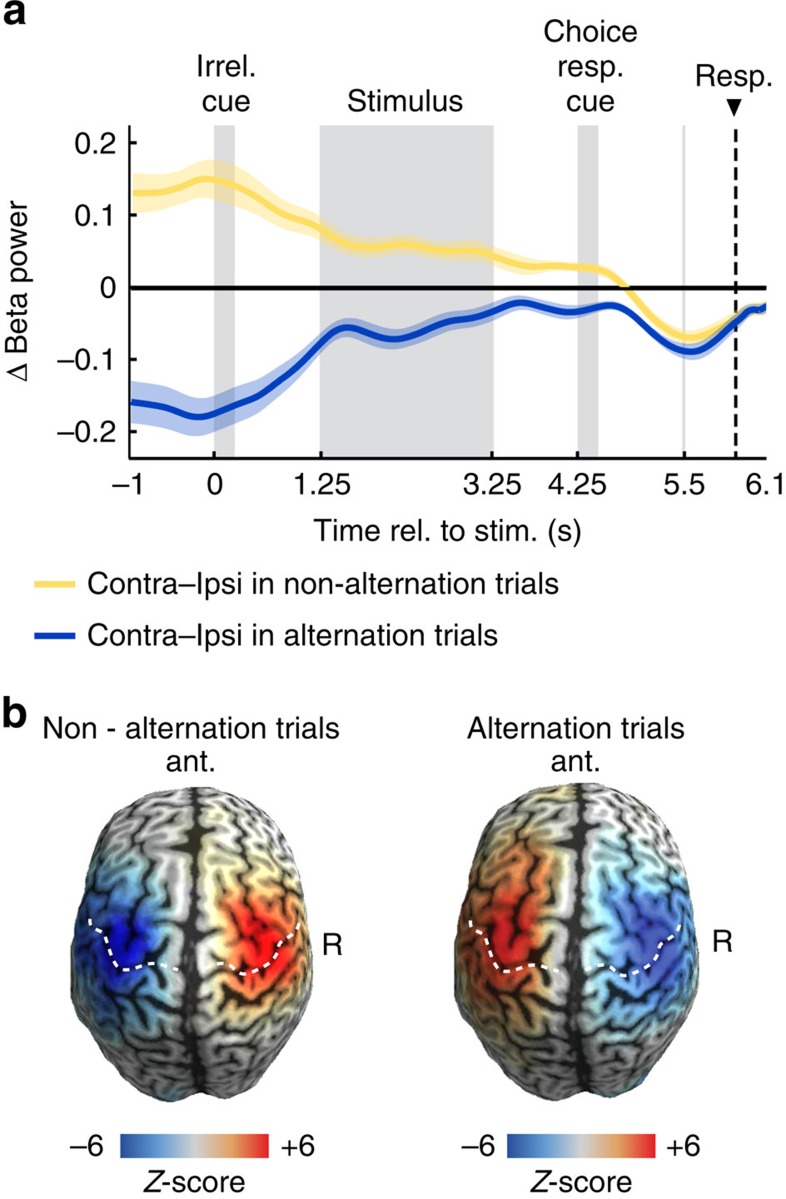
Response-predictive activity in trials with and without alternation. (**a**) Beta lateralization (12–30 Hz; contralateral-ipsilateral to the response) for non-alternation trials (that is, trials where the same button is pressed in the current trial as in the previous trial) is lateralized with a positive sign throughout most of the trial, that is, opposite to the lateralization immediately preceding the button-press at 6.1 s. For alternation trials, the lateralization is negative throughout the entire trial. (**b**) Beta power (12–30 Hz) across the whole cortex during the prestimulus interval (−1 to 1.25 s) for left minus right button-presses (in the current trial) plotted separately for whether the button-press in the current trial is a non-alternation (that is, repetition of the previous button-press) or an alternation with respect to the previous button-press.

**Figure 5:**
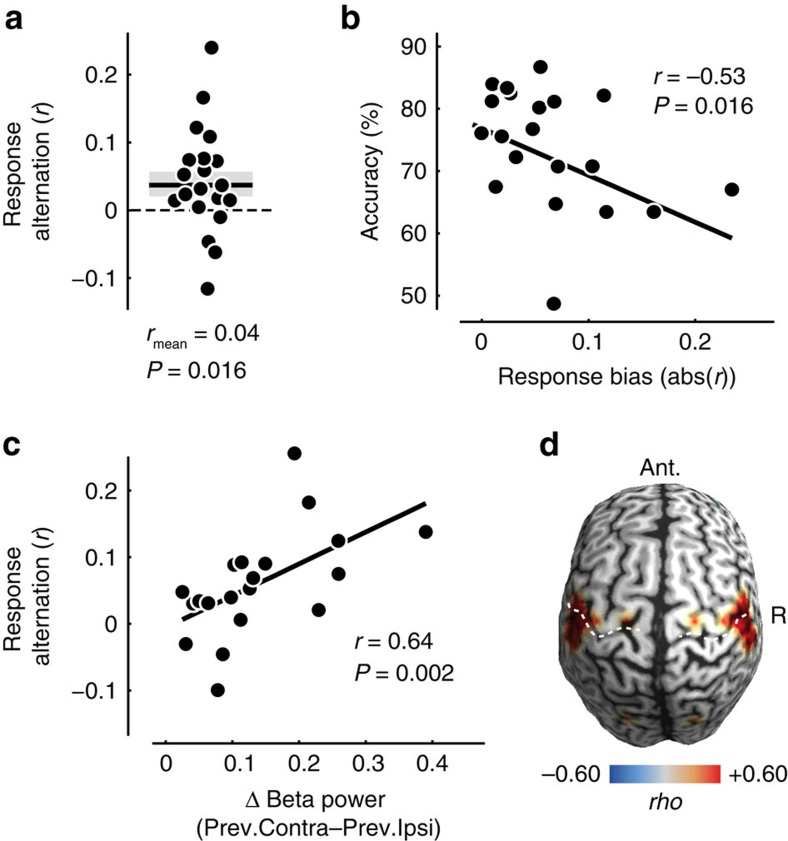
Response alternation and its relation to the beta rebound. (**a**) Each participant's tendency to alternate response hands expressed as Pearson's *r* between opposite hands for consecutive trials. Note that dots are scattered horizontally to avoid overlap. Shaded area denotes SEM. (**b**) Relationship between response bias and choice accuracy across participants. (**c**) Relationship between beta rebound (lateralization with respect to previous button-press from −1 to 1.25 s of the current trial) and response alternation across participants. (**d**) Cortical distribution of the relationship between beta rebound and response alternation across participants. Correlations are masked at *P*<0.05. Note that the image is symmetric across the midline, because the beta rebound was calculated as the lateralization between corresponding points in both hemispheres.

The beta rebound pushes the sensorimotor cortices into a lateralized state opposite to the lateralization before the previous button-press (but see lateralization with respect to current button-press plotted separately for response alternation and non-alternation trials, Fig. 4a,b). We hypothesized that this reversed lateralization following the previous response in combination with the early response-predictive lateralization for the current response may induce a behavioural bias towards response alternations across successive trials. Indeed, participants showed a significant tendency to alternate the response hand from one trial to the next (Fig. 5a, mean *r*=0.04, *P*=0.016, one-tailed one-sample *t*-test; *n*=20). Because our design enabled us to dissociate responses from choices, we could unequivocally dissociate this response alternation bias from the well-known preference to repeat the previous choice^10^,^25^,^26^,^27^, which was also present in our data (mean *r*=0.13, *P*=5.366 × 10^−4^; two-tailed one-sample *t*-test; *n*=20). The response bias also affected overall performance: The stronger the participants' response bias, the worse they performed in the actual motion detection task (Fig. 5b, *r*=−0.53, P=0.016, Spearman correlation; *n*=20).

**Figure 6:**
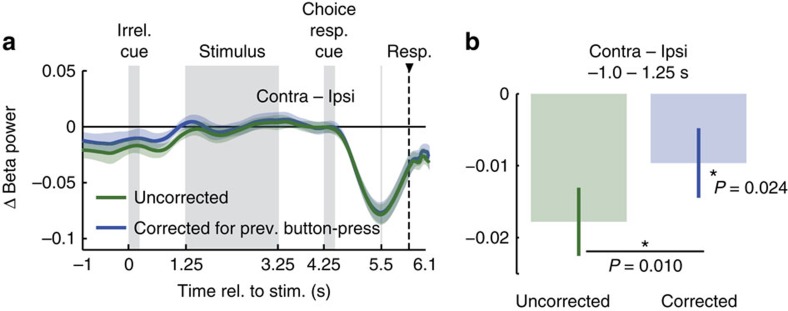
Correcting response-predictive activity for previous responses. (**a**) Time-course of response-predictive beta-lateralization, corrected and un-corrected for previous responses. (**b**) Response-predictive beta-lateralization, corrected and un-corrected for previous responses averaged across the prestimulus window (−1 s to 1.25 s). Error bars show SEM. Correcting for the previous response reduces prestimulus lateralization significantly (*P*=0.010, one-tailed paired permutation test, *n*=20). After correcting for the previous button-press, prestimulus lateralization still predicts the upcoming button-press (*P*=0.024, one-tailed one-sample permutation test, *n*=20).

While the above findings of a long-lasting beta rebound and response alternation suggest a mechanistic link between these two phenomena, they might also merely coexist. Therefore, we sought more direct evidence for a link between these two phenomena. If they were mechanistically related, variance in one variable should explain variance in the other. First, we tested if, across participants, the strength of the beta rebound predicted the tendency to alternate responses. This is what we found (Fig. 5c, *r*=0.64, *P*=0.002, Spearman correlation; *n*=20): the stronger a participant's beta rebound, the more likely the participant was to alternate responses. We repeated this analysis across the entire cortex (Fig. 5d). This revealed that the beta rebound predicted response alternation specifically in regions compatible with sensorimotor cortex and similar to those regions showing maximum pre-response lateralization (Fig. 2b). Second, we tested if the relationship between beta rebound and response alternation also held on the single-trial level. Indeed, we found that the stronger the beta rebound at the beginning of a trial, the more likely were participants to alternate responses on this trial (random effects: *P*=0.021; fixed effects: *P*=0.005; two-tailed one-sample permutation tests on beta rebound averaged in the window −1 to −1.25 s; *n*=20 ). Another third line of evidence suggested a close relation between beta rebound and alternation behaviour: If the response-predictive activity at trial onset (Fig. 2d) reflects the effect of the beta rebound on response behaviour, then removing neural variability due to the beta rebound should reduce the response-predictive effect. To test this, we removed neural variability due to the beta rebound by correcting for the effect of previous responses (see ‘Methods' section). Indeed, we found that this correction significantly reduced the response-predictive effect (Fig. 6a,b, *P*=0.010, one-tailed paired permutation test; *n*=20). This finding provides additional evidence for a mechanistic link between beta rebound and response alternation behaviour.

We next tested if the strength of the beta rebound was modulated by different aspects of the previous trial. We found that only the duration of the preceding inter-trial interval (ITI; *P*<0.001; two-tailed one-sample *t*-test; *n*=20), but not the previous choice, response hand, target presence, accuracy, or reaction time (all *P*>0.05; two-tailed one-sample *t*-tests, all *n*=20) predicted the strength of the following beta-rebound (Supplementary Table 1). Corresponding to this decay of the beta-rebound, also the alternation bias was descriptively weaker and not significant for trials following long (mean *r*=0.019, *P*=0.45, one-tailed one-sample *t*-test; *n*=20) as compared with short (mean *r*=0.052, *P*=0.046, one-tailed one-sample *t*-test; *n*=20) inter-trial intervals (direct comparison *P*=0.21, one-tailed paired *t*-test; *n*=20, Supplementary Fig. 2).

In sum, our findings suggest that the beta rebound drives response-predictive fluctuations of sensorimotor cortex activity at trial onset.

### Spontaneous fluctuations of beta lateralization predict responses

Do also spontaneous fluctuations of motor cortical activity beyond the beta rebound predict responses? In other words, can response variability be explained by prestimulus neural variability—over and above the fact that responses depended on previous responses, and the fact that each response produces a beta rebound? Removing the neural variability due to the beta-rebound allows for also addressing this question. Indeed, we found that even after removing neural variability due to the beta-rebound, motor cortex lateralization at trial onset predicted upcoming responses (*P*=0.024, −1 to 1.25 s, one-tailed one-sample permutation test; *n*=20, Fig. 6a,b). Thus, the response-predictive sensorimotor activity was not limited to the neural aftermath of the previous trial, that is, the beta rebound, but also spontaneous fluctuations unrelated to the previous button-press predicted which button would be pressed 6 s later.

### The effect of choice-contingent response planning

All of the above results held in a situation where choices could be translated into motor responses only after choice formation. Do motor fluctuations also predict responses when choices can be directly mapped onto motor responses? To test this, we recorded MEG during a second decision task in which the choice-response mapping was already cued before the stimulus by swapping the order of the irrelevant and the choice–response cues (choice–response cue for control task: 0–0.25 s).

**Figure 7:**
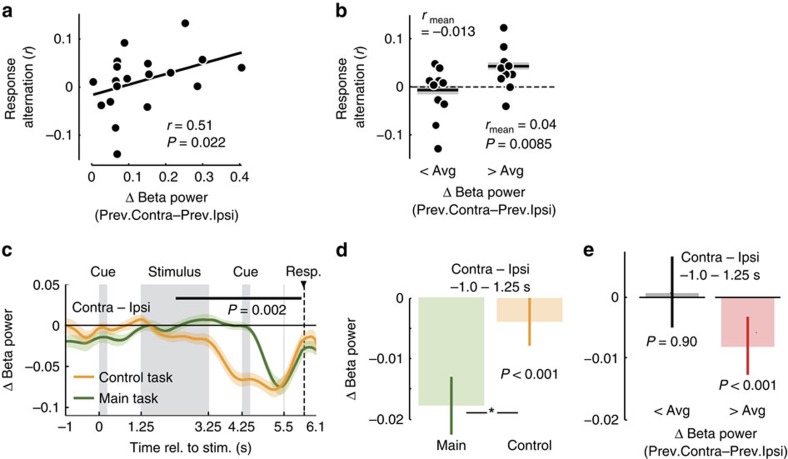
Decision making with known choice-response mapping. (**a**) Relationship between beta rebound (lateralization with respect to previous button-press from −1 to 1.25 s of the current trial) and response alternation across participants. (**b**) Participants' response alternation. Participants are grouped according to above or below median beta rebound. (**c**) Time-course of response-predictive beta activity, that is, of the difference in beta power between hemispheres contra- and ipsilateral to the button-press. The black bar marks significant response-predictive activity in control trials (2.3–6.1 s, *P*=0.002, two-tailed one-sample cluster permutation test, *n*=20). Data from main task re-plotted from Fig. 2c for comparison. (**d**) Lateralization with respect to the upcoming button-press in the prestimulus window of main and control trials. (**e**) For subjects with above median beta rebound, prestimulus lateralization predicts the upcoming response in the control task (*P*<0.001, time window from −1 to 1.25 s, one-tailed one-sample permutation test, *n*=10), whereas this is not possible in subjects with below median beta rebound (*P*=0.90, one-tailed one-sample permutation test, *n*=10).

Motor activity also predicted motor responses in this control task, but weaker. We first focused on the beta rebound as the major source of motor fluctuations. Again, we found evidence for a mechanistic link between beta rebound and response alternation: Across participants, stronger beta rebound significantly predicted stronger response alternation (Fig. 7a, *r*=0.51, *P*=0.022, Spearman correlation; *n*=20), but descriptively the relationship was weaker than for the original task. Correspondingly, participants showed a weaker tendency to alternate responses in the control task, which was only significant in participants with above average beta rebound (Fig. 7b, mean *r*=0.04, *P*=0.0085, one-tailed one-sample *t*-test; *n*=10), but not across the entire sample (all participants: mean *r*=0.015, *P*=0.132, one-tailed one-sample *t*-test; *n*=20). Also the response-predictive effect of early motor lateralization was significantly weaker in the control task than in the original task (Fig. 7d, *P*<0.001, one-tailed permutation test, −1 to 1.25–s; *n*=20), and reached significance only in participants with above-average beta rebound, not in all participants (Fig. 7 e, and Supplementary Fig. 3, −1 to 1.25–s, all participants: *P*=0.18, *n*=20, one-tailed one-sample permutation test; participants with above average beta rebound: *P*<0.001, *n*=10, one-tailed one-sample permutation test). The preference for repeating the same choice as in the previous trial was present in the control task as in original task (mean *r*=0.055, *P*=0.0075, two-tailed one-sample *t*-test).

**Figure 8:**
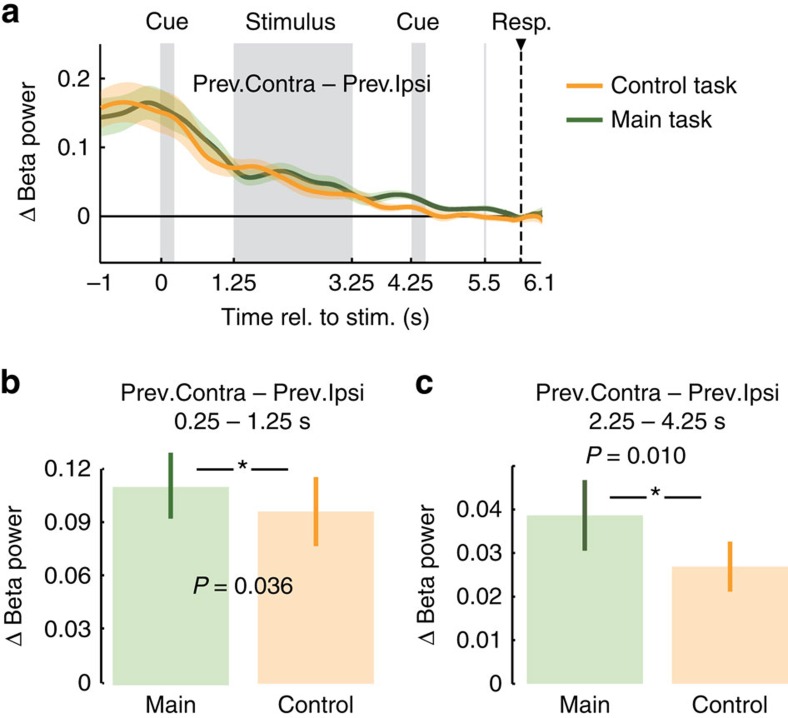
Differences in beta rebound between main and control task. (**a**) Lateralization of beta power with respect to the previous button-press (beta rebound) for main and control tasks across the trial. (**b**) Beta rebound for the main and control task averaged between the end of the first cue (0.25 s) and stimulus onset (1.25 s) are significantly different (*P*=0.036, one-tailed paired permutation test, *n*=20). (**c**) Beta rebound for the main and control task averaged across the second half of the stimulus and the delay period before the second cue (2.25 s–4.25 s) are significantly different (*P*=0.010, one-tailed paired permutation test, *n*=20).

Why was the effect of motor fluctuations on response selection weaker when the choice-response mapping was cued before the stimulus? We hypothesized that this may reflect interference of early response planning with the prestimulus motor lateralization. Indeed, in accordance with previous reports^9^,^10^, for the control task, response-predictive lateralization started already during the stimulus interval (Fig. 7c, 2.3–6.1 s, *P*=0.002, two-tailed one-sample cluster permutation test, *n*=20). Thus, in the control task, subjects already mapped choices onto response plans during decision formation, that is, earlier than in the main task. The possibility to plan responses early on may have decreased the preexistent motor lateralization. To test this hypothesis, we compared the beta rebound between the original and the control task while ruling out confounds due to the different alternation behaviour (Fig. 8a, see ‘Methods' section). As hypothesized, the beta rebound was significantly decreased for the control task in the late stimulus interval and delay before the second cue, that is, during response planning in the control task (Fig. 8c, *P*=0.010, one-tailed paired permutation test, *n*=20). Notably, the beta rebound was also already reduced in the delay interval directly following the early choice-response cue in the control task (Fig. 8b, *P*=0.036, one-tailed paired permutation test, *n*=20), which may reflect the suppression of the beta rebound in preparation of the upcoming response planning or processing of the choice–response cue. Together, these results suggest a reduced response-alternation bias in the control task because upcoming or evolving response planning reduces motor fluctuations caused by previous responses.

## Discussion

Our results provide new insights into sensorimotor decision making on both behavioural and neural levels. We uncovered that a previous motor response can influence sensorimotor decision making. Several factors beyond the present stimulus are known to influence sensorimotor decisions. These factors include neural noise at sensory stages^28^,^29^, top-down factors such as stimulus^10^ and reward^30^ expectations, motor costs associated with response options^31^,^32^,^33^ or sequence effects such as the ‘Gambler's fallacy', that is, the mistaken belief that high event incidence is followed by low incidence and vice versa^27^, or the preference to repeat the previous perceptual choice^10^,^25^,^26^,^27^ that we also observed in the present experiment. The Gambler's fallacy and choice repetition effect are conceptualized on the choice-level, that is, the content of decisions (for example, ‘yes—I saw the target'). In contrast, our results indicate that also previous responses at the level of the motor act (for example, a specific button-press) and independent of previous choices influence which decisions are eventually reported. This unravels a previously unknown decision factor that needs to be accounted for in models of decision making as well as in the analysis and design of decision-making experiments. In fact, our results suggest that, for perceptual decision-making tasks with fixed choice–response mapping, the well-known choice-repetition bias is counteracted by an independent response-alternation bias.

While the demonstrated response-alternation bias is behaviourally detrimental for perceptual decision-making tasks, such as the one at hand, it may be beneficial in specific behavioural contexts. For instance, response alternation may improve sampling of different motor acts to succeed in a task, favoring exploration over exploitation, or it may help prevent motor fatigue.

We identified the post-movement beta-rebound as a strong source of sensorimotor cortex fluctuations that may drive the response-alternation bias. Three lines of evidence support this conclusion. First, subjects with stronger beta-rebound showed stronger response alternation. Second, the strength of beta-rebound predicted the likelihood of response alternation on the single-trial level. Third, removing neuronal variability related to the previous response's beta-rebound reduced the early response-predictive beta lateralization.

Our results accord well with other recent studies that provide converging correlative^34^,^35^,^36^,^37^ and manipulative^38^,^39^ evidence for a causal role of beta-oscillations in motor control. Nevertheless, it remains difficult to pinpoint the exact neural source of the demonstrated alternation behaviour based on the present data alone. First, although we found strongest effects in regions consistent with primary motor cortex and applied source-reconstruction to extract primary motor cortex activity, the spatial resolution of MEG is limited. Thus, other regions such as for example, premotor cortex or somatosensory cortex^40^ may well contribute to the observed effects. Second, only regions with a prominent macroscopic contralateral motor organization were apt to reflect upcoming or past responses in the present experiment. This organization decreases upstream from primary motor cortex, which reduces response-predictive lateralization. Thus, the effects that we observed over motor cortex may in principle be caused by other upstream cortical or subcortical^41^,^42^ regions that encode response specific information without a somatotopic organization. In addition, post-central somatosensory areas might contribute to the observed beta oscillations. Previous research has demonstrated monosynaptic projections from S1 onto motoneurons^43^ and beta coherence between S1 and muscle activity^40^. Yet, S1 stimulation does not elicit or facilitate muscle activity^44^. Thus, the role of S1 in motor control remains unclear. In sum, while our results suggest an intimate relationship of the motor cortical beta rebound and response alternation, the exact cortical mechanisms that drive response alternation remain to be determined. Ultimately, invasive and manipulative approaches will be required to unequivocally show that motor cortex activity itself causes the response-alternation bias. Independent from the exact cortical stage, our results show that a post-response rebound of neural representations of motor responses predicts response alternation in human decision making.

Furthermore, our results show that even beyond the response-related beta-rebound the state of the sensorimotor cortex before decision formation and unrelated to choice content predicts the final decision-making outcome. Previous studies showed that neuronal activity in motor areas reflects upcoming choices during evidence accumulation if choices and responses are inextricably linked^9^,^10^,^16^,^45^. Our finding of response-predictive, but choice-unrelated activity suggests that sensorimotor cortex activity during decision making does not merely reflect the routing of decision-related activity from higher cognitive areas^18^,^46^, but that motor cortex activity itself can act on the resolution of response competition in a distributed network of decision making^12^. As such our results accord well with a growing body of evidence suggesting that motor regions are directly involved in the process of decision making^4^,^5^,^6^,^8^,^11^,^15^,^47^. That said, our results are also well compatible with converging data that suggest a prominent role of frontoparietal association cortices in decision making^1^,^12^,^18^,^46^,^48^,^49^,^50^.

In summary, our results show that not only choice-related neuronal fluctuations but also fluctuations related to the associated motor responses predict sensorimotor decisions.

## Methods

### Participants

Twenty healthy, right-handed volunteers (11 female, mean age 29 years) participated in this study. All had normal or corrected-to-normal vision and received monetary reward for their participation. The study was conducted in accordance with the Declaration of Helsinki, and was approved by the ethics committee of the University of Tuebingen. All participants gave written informed consent before participating.

### Behavioural task

On each trial, participants had to decide whether coherent motion was present in centrally presented dynamic random dot pattern (random dot kinematogram, RDK) and to report their percept (yes/no) by button-press with the left or the right index finger (Fig. 1a, 2-alternative forced choice). The choice- response mapping was newly assigned on each trial by a colour cue (red or green). For the main and control task, this choic–response cue was presented after or before the stimulus, respectively. For temporal symmetry, an irrelevant cue (blue) was presented before or after the stimulus for the main and control task, respectively. Each trial started with a 1.5 s fixation period, followed by the first 0.25 s cue period, a blank 1 s delay, 2 s of stimulus presentation, another 1 s delay, the second 0.25 s cue period, another 1s delay and a brief (33 ms) dimming of the fixation spot, which served as the go-cue to respond. The mean (across subjects) median +/−5/95 percentile (within subject) response times were 0.63 +/− 0.37/1.42 s and 0.62 +/− 0.35/1.38 s for the main and control experiments, respectively. There was no significant difference of response times between right- and left-hand responses for the main or control experiment (both *P*>0.05; permutation test, *n*=20). 250 ms after the response, a brief (100 ms) visual feedback was presented centrally (red or green circle; 2.1 degree diameter; green: correct, red: incorrect). The following ITI was controlled by the participants through their fixation behaviour. The experiment was paused as long as participants did not fixate the central fixation spot or closed their eyes. The pause was indicated by presentation of thin red lines at the edges of the screen. This resulted in variable inter trial intervals with a median duration of 1,290 ms. Participants were instructed to blink only during the ITI. Subjects completed 240 trials of the main task and 240 trials of the control task in two consecutive recording sessions. In addition, participants performed 240 trials (cued task), for which participants did not have to make a decision about the stimulus but received explicit instructions which button to press on each trial. Furthermore, for another 80 trials (passive task) participants had to press no button at all, but were instructed to passively view the stimulus. Cued and passive task trials were not analysed for the present study. All tasks were randomly interleaved. Before the recording, participants practiced the task for at least 45 min.

### Stimuli

Dynamic random dot patterns were presented for 2 s and consisted of 1,500 white dots (dot diameter: 0.12 deg) on a black background, moving at 10 deg s^−1^ according to the ‘random direction, different rule'^51^ in a circular aperture of 8.5 deg diameter. For each participant, there were exactly two stimuli, both presented half of the trials: in the noise-only stimulus, there was no coherent motion, whereas in the target stimulus, a fraction of dots moved coherently downwards. Motion coherence of target stimuli was titrated to each participant's perceptual threshold employing a staircase procedure with 280 trials and a Weibull function fit (average target motion coherence: 9%). All colour cues had the same luminance (14 cd m^−^^2^) and size (0.85 deg diameter). Choice-response mapping was assigned as follows: red: Yes → right, No → left, green: Yes → left, No → right, blue: uninformative.

### Setup and neurophysiological recordings

We recorded the MEG (Omega 2000, CTF Systems, Inc., Port Coquitlam, Canada ) with 275 channels at a sampling rate of 2,343.75 Hz in a magnetically shielded chamber. Participants were comfortably seated upright in a dark room. Stimuli were projected onto a screen at a viewing distance of 55 cm using a hue and luminance calibrated liquid crystal display projector (Sanyo PLC-XP41, Moriguchi, Japan) at 60 Hz refresh rate. Stimuli were constructed offline and presented using the Presentation software (NeuroBehavioral Systems, Albany, CA, USA). In addition to the MEG, we recorded the electrooculogram and electrocardigram for offline artefact rejection.

### Eye movement recordings

Throughout the experiment, we recorded the participants' eye movements with a video-based eye-tracker (EyeLink 1000, SR Research, Ottawa, Canada). This ensured continuous fixation and allowed participants to conveniently control the length of the ITI.

### Structural MRI

For source reconstruction based on each participant's individual anatomy, we recorded structural T1-weighted MRIs of each participant (echo time (TE)=2.18 ms, repetition time (TR)=2.3 ms, longitudinal relaxation time (T1)=1.1 ms, flip angle=9°, 192 slices, voxel size 1 × 1 × 1 mm^3^) with a Siemens 3T Tim Trio scanner and a 32 channel Head Coil.

### MEG preprocessing

MEG data were downsampled to 1,000 Hz and high-pass filtered at 4 Hz (two-pass Butterworth filter, filter order 6). Line noise and its harmonics were notched out (49.5–50.5 Hz, 99.5–100.5 Hz, 149.5–150.5 Hz ;199.5–200.5 Hz, 249.5–250.5 Hz, 299.5–300.5 Hz, 349.5–350.5 Hz two-pass Butterworth filter, filter order 4), and after careful visual inspection of the respective signals, trials with eye blinks, saccades, strong muscle artifacts, or signal jumps were excluded from further analyses (on average 20% and 18% of all trials for the main and control task, respectively).

### Source analysis

We used adaptive linear spatial filtering (beamforming)^52^,^53^ to estimate neural population signals at the source level. We used frequency-domain beamforming dynamical imaging of coherent sources (DICS)^52^ to investigate the cortex-wide distribution of response-predictive beta-band activity before the button-press. We used time-domain beamforming linearly constrained minimum variance (LCMV)^53^ to analyse the dynamics of frequency-specific neural activity in motor cortex.

The implementation details of the beamformer were as follows: for each time *t*, frequency *f* (for frequency-domain beamforming) and source location *r*, three orthogonal filters (**Â***=*[*A*_*x*_*, A*_*y*_*, A*_*z*_]; one for each spatial dimension) were computed that pass activity from location **r** with unit gain, while maximally suppressing activity from all other sources:


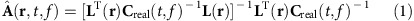


Here, **L**(**r**) is a matrix whose columns are the leadfields of three orthogonal dipoles at source location **r**, and **C**_**real**_ denotes the real part of the complex cross-spectral-density matrix for the data at frequency *f* and time *t*, and ^T^ indicates the matrix transpose. For time-domain beamforming, filters are not frequency dependent and **C**_real_ denotes the covariance matrix of the sensor-level signals. We derived a joint filter for all contrasted conditions.

We linearly combined the three filters to a single filter pointing in the direction of maximal variance, that is, the dominant dipole orientation. To this end, the filters were weighted with the first eigenvectors' elements (the eigenvector with the largest eigenvalue of the real part of the cross-spectral-density or covariance matrix at the source location **r**):


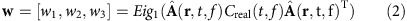



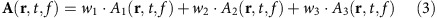


To derive the complex source estimates (frequency-domain beamforming), the complex frequency-domain data was multiplied with the real-valued filter:


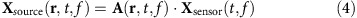


where **X**_sensor_(*t,f*) is the frequency-domain representation at time *t* and frequency *f* at the sensor level and **X**_source_(**r***,t,f*) is the corresponding source signal at location **r**. For time-domain beamforming, **X**_sensor_ and **X**_source_ denote the sensor-level and source-level timecourses, respectively.

### Source locations

To investigate the cortical distribution of choice predictive neuronal activity before the button-press (Fig. 2b), we estimated neuronal activity at 457 source locations that homogeneously covered the space at ∼0.7 cm beneath the skull with a spacing of ∼1.25 cm. This coverage is well adapted to the spatial resolution of MEG, samples sources with high signal-to-noise ratio (SNR) close to the sensors, and covers a large part of the cortex.

Furthermore, we reconstructed neuronal activity specific to the button-press near the hand representation of left and right primary motor cortex. We visually inspected each participant's cortical map of the contrast between contra- and ipsilateral button-presses in main, control and cued tasks in the time-window from 4.5 to 5.5 s and the frequency range from 12 to 30 Hz. For each participant, we selected the local spatial maximum of this functional contrast closest to the anatomical hand representation, that is, the ‘handknob' of the precentral gyrus.

### Physical forward model for source analysis

For all source analyses, we computed individual physical forward models (leadfields). To match participants, we nonlinearly transformed source locations defined in standard Montreal Neurological Institute (MNI) space into individual head space using the participants' individual structural magnetic resonance image (MRI). We aligned the MEG sensors to the head geometry based on three fiducial points (nasion, left and right ear, registered during the MEG acquisition by three head localization coils). For each participant, we derived the physical relation between sources and sensors using a single shell model^54^ that was computed based on the segmentation of each participants structural MRI.

### Spectral analysis

For time-frequency analyses of neuronal activity (Figs 2a and 3a), we source-reconstructed broad-band neuronal activity using time-domain beamforming and employed a sliding window multi-taper Fourier analysis (window size: 250 ms, step size: 20 ms, 8 Hz smoothing, 3 discrete prolate spheroidal sequences (DPSS) tapers). To account for variable response times, we computed two time-frequency transforms: first, with data aligned to the stimulus, and second, with data aligned to the button-press. These time-frequency transforms were concatenated according to the average response time. Power was quantified as the per cent change of power relative to the average pre-cue baseline.

To image the cortical distribution of response-predictive beta-band activity directly preceding the response, we derived the sensor-level cross-spectral density matrix for frequency-domain beamforming using multi-taper Fourier analysis (4.5–5.5 s, 12–30 Hz, 17 discrete prolate spheroidal sequences tapers).

To investigate the time-course of source-reconstructed beta-band activity, we band-pass-filtered the sensor-level MEG data in the time-domain (12–30 Hz; two-pass Butterworth filter, filter order 4), applied time-domain beamforming, applied the Hilbert transform, and smoothed power time-courses with a 500 ms (full-width at half-maximum) Hanning window. Finally, all time-courses were normalized by the average across time and trials.

### Response-predictive activity

To isolate neuronal activity that predicted the specific upcoming response (left or right hand), we contrasted power in motor cortex contra- and ipsilateral to the response hand (Figs 2d, 6, 7c,d). This contrast isolates effector-specific signals and discards other unrelated neuronal variance providing a specific proxy on neuronal activity involved in decision formation and motor execution^9^,^21^,^22^. This contrast can be formalized as:


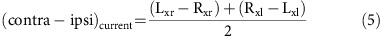


where L and R stand for the neuronal activity measured in the left and right hemisphere, respectively, and the first and second subscripts denote the previous and current response hand, respectively. r, l, and × denote right, left and either response hand, respectively. Thus, 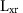 denotes the left hemispheric activity measured for trials with left- or right-hand button-press on the previous trial and right-hand button-press on the current trial. The left and right bracketed terms in equation (5) correspond to neural activity contralateral–ipsilateral to current right and left-hand button-presses, respectively.

### Beta rebound

To estimate the response-specific effect of the previous button-press on the current trial, that is, the beta-rebound, we contrasted power in motor cortex contra- and ipsilateral to the previous trial's button-press (Figs 3c, 8a–c):


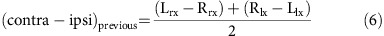


The left and right bracketed terms in equation (6) correspond to neural activity contralateral–ipsilateral to previous right and left-hand button-presses, respectively. To quantify the response-specific beta-rebound for each subject, we averaged lateralization relative to the previous response from −1 to 1.25 s of the current trial.

### Statistical assessment of lateralization

To assess statistical significance of response-specific lateralization across time and frequency (Fig. 2a) or across time (Figs 2c,d and 3b,c and 7c), we calculated cluster permutation statistics that account for multiple comparisons with a first-level threshold of *P*=0.05 (two-tailed) and 1,000 subject-level permutations^55^,^56^. For all contrasts tested on specific time windows (Figs 2e, 3e, 6b, 7d,e and 8b,c), we employed permutation statistics on un-smoothed data with 1,000 subject-level permutations. One often employed time-window was from −1 to 1.25 s (Figs 2e, 3e, 6b, 7d,e). We used this window, because this period includes the entire prestimulus interval that well matches the extent of the early response-predictive beta lateralization (Fig. 2d). All statistics were computed across subjects (random effects) with two-tailed tests unless noted otherwise.

### Correction for previous responses

To investigate the beta rebound's contribution to the early response-predictive activity, we computed the lateralization relative to the current trial's response corrected for the previous response (Fig. 6):


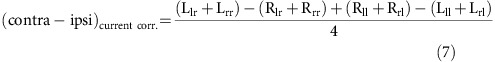


The effect of previous responses is corrected for by computing the responses contralateral and ipsilateral to the current response averaged across trials with equal weighting across both possible previous responses (the four bracketed terms in equation (7)). In other words, we replace the four numerator terms in equation (5) with the same terms balanced for the previous response. By re-ordering equation (7) it becomes evident that this balancing removes the previous trial's effect:


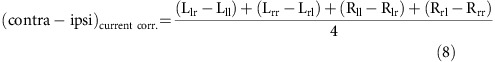


Each of the four bracketed numerator terms in equation (8) isolates the effect of the current response (contralateral–ipsilateral) and subtracts out the effect of a specific previous response for a specific hemisphere. By removing the effect of previous responses, this correction removes the neuronal variability specific to the previous response, that is, the beta rebound. We employed this correction not only to test if the beta rebound contributed to the early response-predictive activity, but also to test if spontaneous, that is, beta-rebound independent, fluctuations of motor cortex lateralization predict responses.

We applied the same correction also when comparing the size of the beta rebound between main and control tasks (Fig. 8a–c). This allowed us to rule out potential confounding by different alternation behaviour across tasks (for example, less alternation trials for the control task) because correcting for the previous response is equivalent to correcting for alternation behaviour. Again, this becomes evident by re-ordering equation (7) accordingly:


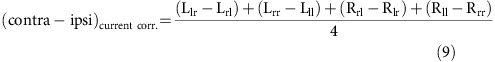


Now, each of the four bracketed terms in equation (9) isolates the effect of the current response (contralateral–ipsilateral) and subtracts out the effect of the previous response being the same or different from the previous response.

### Correlation analyses

To quantify relations between nominal behavioural variables (responses 'left' or ‘right' on current and previous trials) we used Pearson's correlation coefficient for binary variables (Phi coefficient). To assess statistical significance of correlations, we Fisher-z-transformed subjects' *r*-values and applied two-tailed t-statistics across subjects unless noted otherwise.

To test if different aspects of the previous trial modulated the strength of the beta rebound we performed a multivariate partial correlation analysis, with the predictors previous choice, previous response hand, previous target presence, previous accuracy, previous reaction time, and ITI duration following the previous response. For each subject, partial correlation was performed across trials and the significance of predictors was assessed using a two-tailed t-statistics of the Fisher-z-transformed *r*-values across subjects.

To quantify the relation between each participant's beta rebound and tendency to alternate responses on the subject level, we computed Spearman's rank correlation across subjects (Fig. 5c). We used the same approach to test for each cortical region, how its beta rebound predicted response alternation (Fig. 5d). To test if the strength of the beta rebound also predicted the tendency to alternate responses on the single-trial level we either tested for a difference of the beta-rebound between alternation and non-alternation trials across subjects (random effects), or we tested for a difference of the beta-rebound between alternation and non-alternation trials pooling all trials across subjects (fixed effects). For both approaches, we employed permutations statistics and we z-scored each subject's single-trial beta-rebound data. Thus, both single-trial correlation analyses (random and fixed effects) were orthogonal to the subject-level correlation analysis.

To test if the tendency to alternate responses and accuracy were related, we calculated Pearson's correlation across participants.

All analyses were performed in MATLAB (MathWorks Inc., Natick, USA) using custom software and the Fieldtrip toolbox^57^.

### Data availability

The data that support the findings of this study are available from the corresponding authors upon request.

## Additional information

**How to cite this article**: Pape, A.-A. *et al*. Motor cortex activity predicts response alternation during sensorimotor decisions. *Nat. Commun.* **7,** 13098 doi: 10.1038/ncomms13098 (2016).

## Supplementary Material

Supplementary Information Supplementary Figures 1 - 3 and Supplementary Table 1
