# Supplementary material for: Motor cortex activity predicts response alternation during sensorimotor decisions
Source: Nat Commun. 2016 Oct 7;7:13098. doi: 10.1038/ncomms13098 (PMC5059771; doi:10.1038/ncomms13098)
Supplement: Supplementary Information — Supplementary Figures 1 - 3 and Supplementary Table 1 [file ncomms13098-s1.pdf]

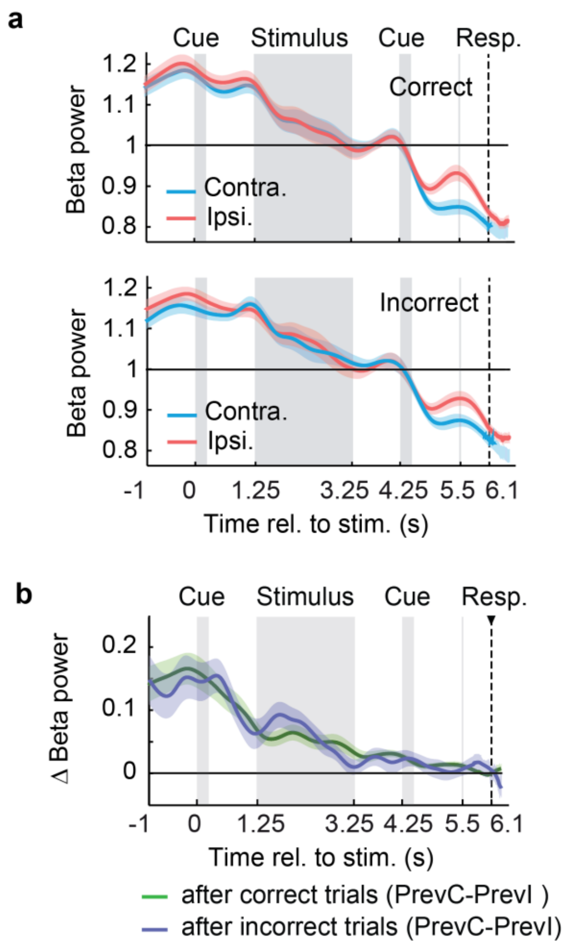

Supplementary Figure 1. Accuracy has no effect on neural variables (**a**) Beta power contra- and ipsilateral to the buttonpress plotted separately for correct (upper) and incorrect (lower) choices on the current trial. Contralateral beta power is lower than ipsilateral beta power in the prestimulus period in both trial categories, i.e. independent from accuracy. (**b**) Beta rebound (i.e. beta power lateralization calculated with respect to the previous button-press) following correct and incorrect choices.

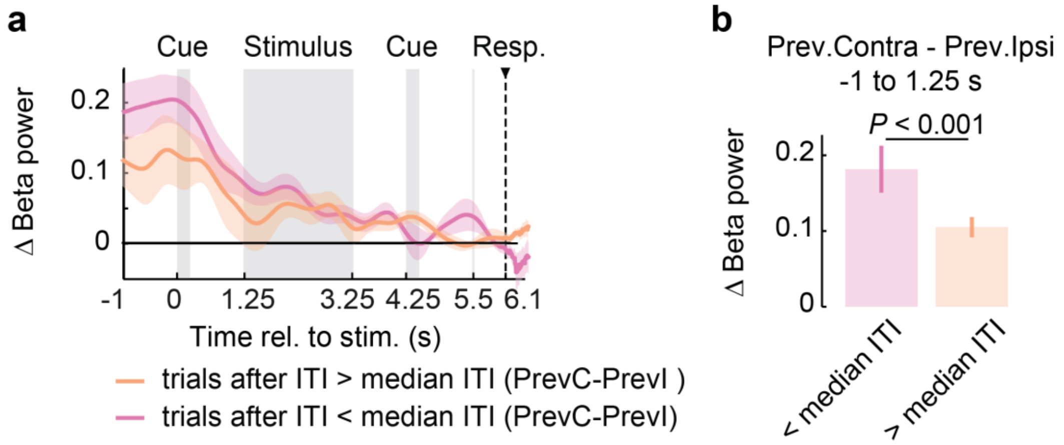

Supplementary Figure 2. Size of beta rebound after long and short intertrial intervals

Participants controlled the length of intertrial intervals (ITI) with their fixation behavior. As the beta rebound occurs locked to the previous button-press, it should decay over time, i.e. it should be smaller for longer ITIs. (**a**) Time-course of the beta rebound for trials following short (< median) and long (> median) ITIs (median ITI = 1290ms). (**b**) As hypothesized, the beta-rebound in the prestimulus interval was significantly smaller following long as compared to short ITIs ($P < 0.001$, two-tailed paired permutation test, $n = 20$). This may lead to weaker alternation behavior for long ITIs. Indeed, while for short ITIs participants showed significant response alternation (mean $r = 0.052$, $P = 0.046$, one-tailed one-sample T-test, $n = 20$), for long ITIs, response alternation was weaker and not significant (mean $r = 0.019$, $P = 0.45$, one-tailed one-sample T-test, $n = 20$). However, a direct comparison between short and long ITIs did not reach statistical significance ($P = 0.21$, one-tailed paired T-test, $n = 20$).

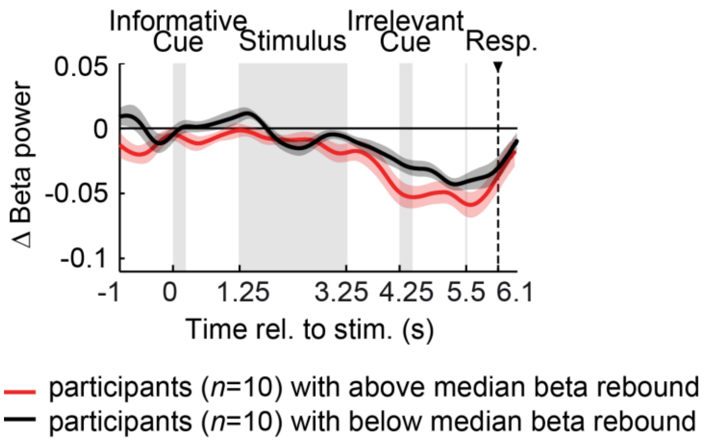

Supplementary Figure 3. Response-predictive activity in control trials

To investigate if the same trend of response-predictive prestimulus lateralization holds for the control task, we split the participants according to the size of their beta rebound. Here, we show the time-course of lateralization for subjects with above and below median beta-rebound separately.

**Factor**	**Mean*(r)*-value**	***P*-value**	***n***
Prev. response hand<br>(left or right hand)	0.067	0.24	20
Prev. choice<br>(yes or no)	0.012	0.65	20
Prev. stimulus<br>(coherent motion present or absent)	0.006	0.78	20
Prev. accuracy<br>(correct or incorrect choice)	0.001	0.97	20
Prev. RT<br>(time from Go cue to button-press)	-0.010	0.66	20
ITI duration	-0.162	< 0.0001	20

Supplementary Table 1. Impact of prev trial parameters on size of the beta rebound

Results of a partial multivariate correlation between the size of the beta rebound (beta-power contralateral minus ipsilateral to the previous button-press in the time window -1 to 1.25s of the current trial) and 5 parameters of the previous trial, calculated in each participant and tested for significance across participants. Only the ITI duration was significantly correlated with the strength of the beta rebound. This effect is further quantified in Supplementary Figure 2.
